# Traceability of animal protein byproducts in ruminants by multivariate analysis of isotope ratio mass spectrometry to prevent transmission of prion diseases

**DOI:** 10.1590/1678-9199-JVATITD-1487-18

**Published:** 2019-05-13

**Authors:** Rui Seabra Ferreira, Daniela Alessandra Fossato da Silva, Natália Perussi Biscola, Maria Márcia Pereira Sartori, Juliana Célia Denadai, André Mendes Jorge, Lucilene Delazari dos Santos, Benedito Barraviera

**Affiliations:** 1 Center for the Study of Venoms and Venomous Animals (CEVAP), UNESP - São Paulo State University, Botucatu, SP, Brazil.; 2 Botucatu Medical School, UNESP - São Paulo State University, Botucatu, SP, Brazil.; 3 College of Agricultural Sciences (FCA), UNESP - São Paulo State University, Botucatu, SP, Brazil.; 4 Stable Isotopes Center (CIE), UNESP - São Paulo State University, Botucatu, SP, Brazil.; 5 College of Veterinary Medicine and Animal Husbandry (FMVZ), UNESP - São Paulo State University, Botucatu, SP, Brazil.; 6 Department of Neurology, David Geffen School of Medicine at UCLA, Los Angeles, CA, USA.

**Keywords:** prion diseases, monitoring, bovine spongiform, encephalopathy, stable isotopes

## Abstract

**Background:**

Ruminant feed containing animal byproduct proteins (ABPs) is prohibited in many countries due to its risk of transmitting prion diseases (PD). In most cases the entire herd is sacrificed, which causes great harm to the producer countries by preventing their exportation of ruminant derived-products.

**Methods:**

We used stable isotope ratio mass spectrometry (IRMS) of carbon (^13^C/^12^C) and nitrogen (^15^N/^14^N) to trace the animal protein in the blood of 15 buffaloes (*Bubalus bubalis*) divided into three experimental groups: 1 - received only vegetable protein (VP) during 117 days; 2 - received animal and vegetable protein (AVP); and 3 - received animal and vegetable protein with animal protein subsequently removed (AVPR). Groups 2 and 3 received diets containing 13.7% bovine meat and bone meal (MBM) added to a vegetable diet (from days 21-117 in the AVP group and until day 47 in the AVPR group, when MBM was removed).

**Results:**

On the 36th day, differences were detectable in the feeding profile (p <0.01) among the three experimental groups, which remained for a further 49 days (85th day). The AVPR group showed isotopic rate reversibility on the 110th day by presenting values similar to those in the control group (VP) (p> 0.05), indicating that it took 63 days to eliminate MBM in this group. Total atoms exchange (> 95%) of ^13^C and ^15^N was observed through incorporation of the diet into the AVP and AVPR groups.

**Conclusions:**

IRMS is an accurate and sensitive technique for tracing the feeding profile of ruminants through blood analysis, thus enabling investigation of ABP use.

## Background

Prion diseases (PD) are a fatal group of neurodegenerative disorders that include Creutzfeldt-Jakob disease (CJD), kuru, scrapie, chronic wasting disease, and bovine spongiform encephalopathy (BSE) [1-4]. Use of animal byproduct proteins (ABPs) in ruminant feed is prohibited in most countries due to the risk of PD transmission [5,6].

The lack of methods that specifically identify the use of mammalian processed animal proteins (PAPs) to feed ruminants led to the introduction of a ban of PAPs for all farmed animals [7] by amending Regulation 999/2001 [8], through Commission Regulation 1234/2003 [9].

Except for the consumption of ABPs, epidemiological studies have failed to identify other specific risk factors for prion diseases. However, experiments have demonstrated BSE and scrapie transmission by blood transfusion [10,11]. Thus, PD monitoring becomes crucial and is dependent on accurate diagnosis, which still represents a unique challenge in the development of novel assays to explore the prion protein complex [3].

Furthermore, diagnostic tests for PD are costly and can be performed only when the animal shows symptoms. In most cases the entire herd is sacrificed, which causes great harm to the producers and their countries by banning them from exporting their products. Therefore, the search for accurate methods to detect previous animal protein intake is essential, since they can imply how and where the animals were raised, denoting prohibited and/or fraudulent practices, thereby ensuring the quality of the meat consumed [4].

Methods such as DNA Hybridization, "Enzyme Linked Immuno Sorbent Assay (ELISA), and Polymerase Chain Reaction (PCR) were employed to identify ABPs in diets supplied to ruminants [12,13]. However, these techniques are not able to detect the presence of animal protein in final products such as meat, blood, eggs, milk, serum samples, and others.

Accurate methods are necessary to identify previous animal protein intake because they can show how and where the animals were raised, determine whether there is a risk for human consumption and, finally, select animals suitable for exploitation by the pharmaceutical industry [4,14-16].

Recently, the measurement of δ^13^C and δ^15^N using isotope-ratio mass spectrometry (IRMS) has been used successfully in the processes of disease control, authentication and certification of animal products [17-19], traceability [4,20,21] and evaluation of conventional and organic production systems for beef [17,22,23].

Traceability investigation through IRMS starts at its main food source, which includes plants of C_3_ photosynthetic cycle (e.g. rice, wheat, barley, alfalfa, peanut, cotton, etc.), with an average δ^13^C isotopic enrichment relative to ^13^C of = -28 ‰, and plants of C_4_ photosynthetic cycle (e.g., sugarcane, corn, tropical grasses, etc.), with a mean δ^13^C value of -12 ‰ ([24,25]. Stable isotopes are used as dietary markers because they are rapidly replaced in tissues by dietary isotopes [26]. It was possible to detect bovine meat-and-bone meal (MBM) in egg yolk and egg albumen of laying birds, even with the inclusion of other ingredients such as vegetables and yeasts [4,27,28].

This study was designed to detect previous ABP intake by determining the stable isotope ratios for carbon (^13^C/^12^C) and nitrogen (^15^N/^14^N) in ruminant blood in order to prevent the feeding of humans with meat susceptible to prion contamination, and to select animals that would provide biological samples to pharmaceutical companies.

## Material and methods

### Animals and experimental diets

Fifteen Murrah buffaloes *(Bubalus bubalis),* aged one year and weighing approximately 200 kg, were fed vegetable-based diet for 20 days to achieve homogenization of ^13^C and ^15^N isotopic values.

Thus animals were divided into three experimental groups: one control group (Vegetable Protein: VP; n = 4), which continued with the starter diet (vegetable-based diet only) for the 117 days of the experiment (days 0-117); and two treated groups (Animal and Vegetable Protein: AVP, n = 6/group; and Animal and Vegetable Protein Removal: AVPR, n = 5/group) that from the 21^th^ day were fed diets containing 13.7% bovine meat and bone meal (MBM; Mondelli^®^ commercial feed - humidity, 8%; crude protein, 45% and ether extract, 6%). The AVPR group was fed until the 47^th^ day (day 21-47), when the MBM was removed.

All the animals were microchipped and received Coast-cross hay (88.96% dry matter with 92.12% organic matter, 11.45% crude protein, 1.75% ether extract, 30.76% crude fiber, 48.16% nitrogen-free extract, and 7.88% ash) and water *ad libitum* throughout experimental period.

### Ethics statement

This study was conducted in accordance with the Ethical Principles in Animal Research of the Brazilian College of Animal Experimentation and was approved by the Ethics Committee for Animal Experimentation (protocol nº 78/ 2009) of the College of Veterinary Medicine and Animal Husbandry (FMVZ-UNESP), Brazil. Animal welfare was respected throughout experimental period, in accordance with the “five freedoms” defined by the Farm Animal Welfare Council.

At the end of the experiment, the animals from the animal and vegetable protein (AVP) and the animal and vegetable protein removal (AVPR) groups were euthanized following the approved ethical protocol.

### Serum preparation and δ ^13^ C and δ ^15^ N analyses by stable isotope-ratio mass spectrometry (IRMS) 

Blood samples were drawn from the animals’ jugular veins twice a week, from 8:00 am to 10:00 am, collected in 10 mL Vacutainer^®^ tubes of 10 mL and centrifuged at 1,000xg for 30 min at 4ºC. Then 1 mL of serum was lyophilized and stored in microtubes at -20ºC. Lyophilized serum weights of approximately 50-70 µg for ^13^C and 500-600 µg for ^15^N were placed in tin capsules for analysis of ^13^C and ^15^N. Subsequently, the capsules were stored in Elisa microplates and maintained at 4ºC until the time of the isotopic analysis.

Isotopic ratios of ^13^C/^12^C and ^15^N/^14^N were measured in the mass spectrometer Delta V Advantage Isotope Ratio MS (Thermo Scientific^®^). Isotopic ratio values were expressed as delta per thousand (δ) relative to the international standards Pee Dee Belemnite (PDB) for ^13^C and atmospheric air nitrogen for ^15^N, according to equation 1 [25,29]:

$$\delta^{13}{∁}_{\left(sample,\;\;\;standard\right)}=\;\left[\left(\frac{R_{sample}}{R_{standard}}\right)-1\right]\;10^{3}$$ (1)

Where: δ^13^C = relative enrichment of the ^13^C/^12^C ratio in the sample relative to the PDB standard; *R* = isotopic ratio (^13^C/^12^C) of the sample and standard.

### Percentage of exchanged atoms

The percentage of exchanged atoms in serum was determined according to equation 2 [30]:

$$F=1-\;e^{-kt}$$ (2)

Where:

F represents the fraction of exchanged atoms, reliable when more than 95%; t is the total time of experiment; k is the *turnover.*

### Data analysis

The data were subjected to univariate statistical analysis (ANOVA and complemented with Tukey's test), for each day of collection. The period that presented the same characteristic was grouped, forming three groups: one that did not present difference between the treatments; a second that differed from the control group; and a third that differed from the treatment using animal protein. Each response group was applied to principal components analysis (PCA) and discriminate analysis. Values were considered significant when p <0.05.

For evaluation of diet incorporation behavior as a function of time, data were adjusted by linear and nonlinear (polynomial and first order exponential) functions.

### Results and discussion

**Figure 1.**
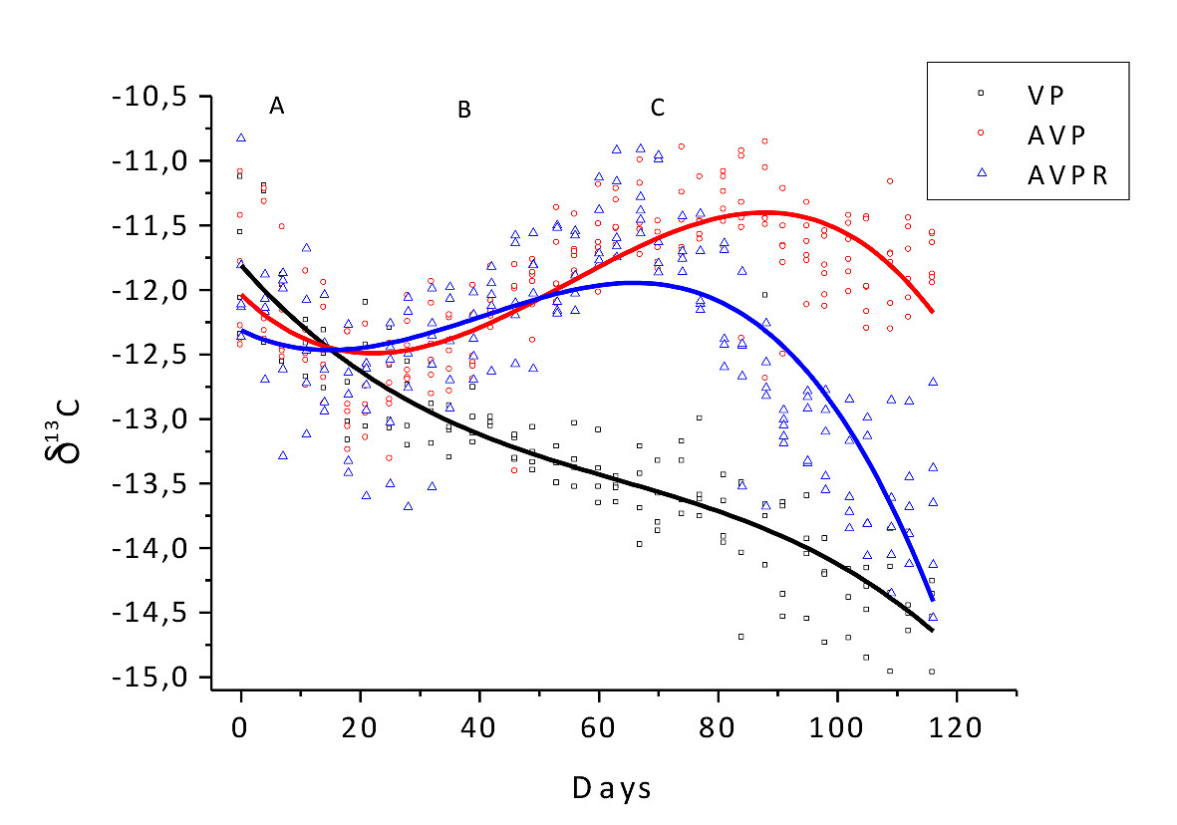
Animal feed δ^13^C measurement. **A:** adaptation period; **B:** period in which the AVP and AVPR groups received diet with animal protein; **C:** period in which the AVPR group received a strictly vegetable diet.

**Figure 2.**
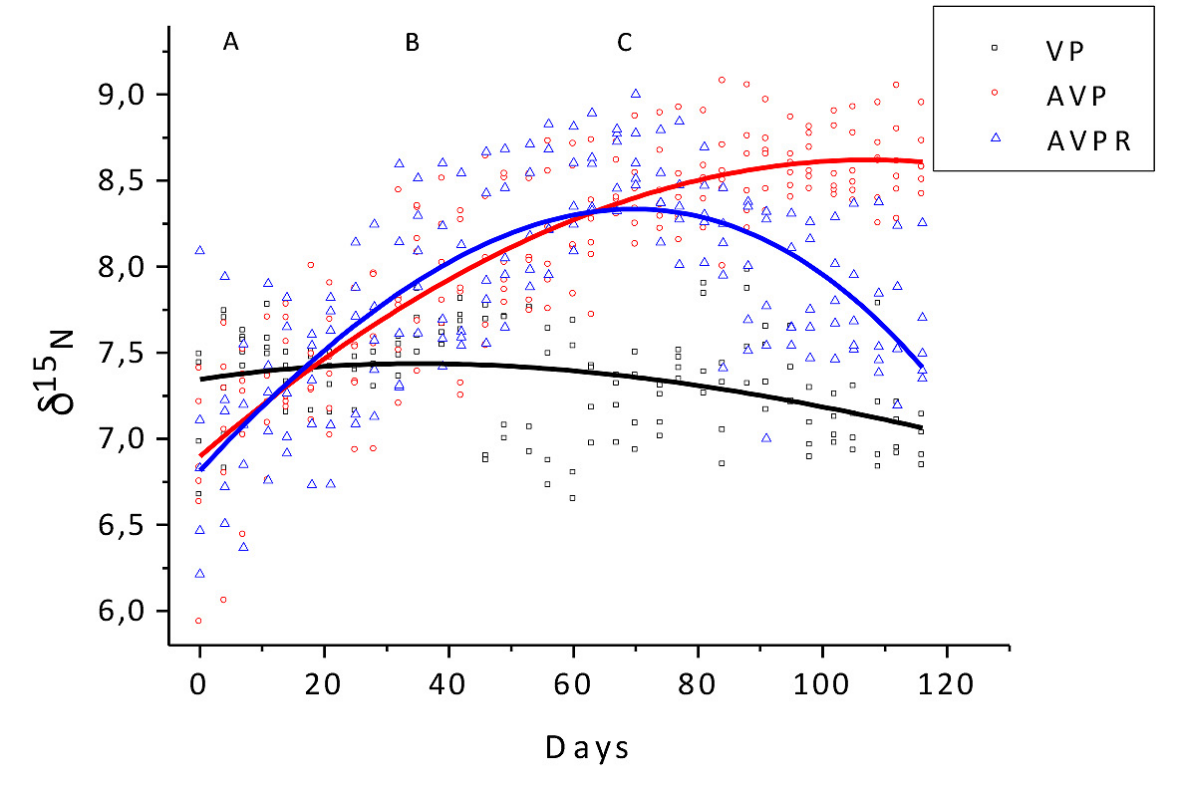
Animal feed δ^15^N measurement. **A:** adaptation period; **B:** period in which the AVP and AVPR groups received diet with animal protein; **C:** period in which the AVPR group received a strictly vegetable diet.

Homogenization of ^13^C and ^15^N isotopic values was possible only by administering the **v**egetable-based diet during the first 20 days to the three experimental groups despite not observing the total exchange of atoms (<95%) in this period (Figures 1 and 2). It was attributable to an isotopic ratio from an animal origin diet outcome based on the *Brachiaria* genus inserted in a system of feeding the photosynthetic cycle C_4_ plants (where δ^13^C varies from -9 ‰ to -16 ‰) and evidenced by a δ^13^C value reduction of 2.5 ‰ in this period (δ^13^C = -12 ‰ to -14.5 ‰), but still encompassing C_4_ values (Figure 1).

However, the isotopic value of ^15^N did not change significantly during the adaptation period (Figure 2), indicating that both source and experimental diets were similar. The ^15^N/^14^N isotopic ratio does not depend on the photosynthetic cycle, but rather on the fixation mode due to the incorporation of nitrogenous compounds of the soil. The ^15^N values can suffer interference from soil type, leguminous plants in the diet and animal metabolic factors [31].

After the adaptation period, a variation in the δ^13^C and δ^15^N isotope values was observed in animals’ serum due to MBM inclusion in the diet remaining in the range of plant values of the C_4_ photosynthetic cycle (Figure 1). This finding verified the isotopic analysis signature of experimental diet that presents a higher percentage of C_4_ plants than C_3_, in addition to MBM, which according to Denadai et al. [27,28], shows a C_4_ isotopic value of -12.82.

**Figure 3.**
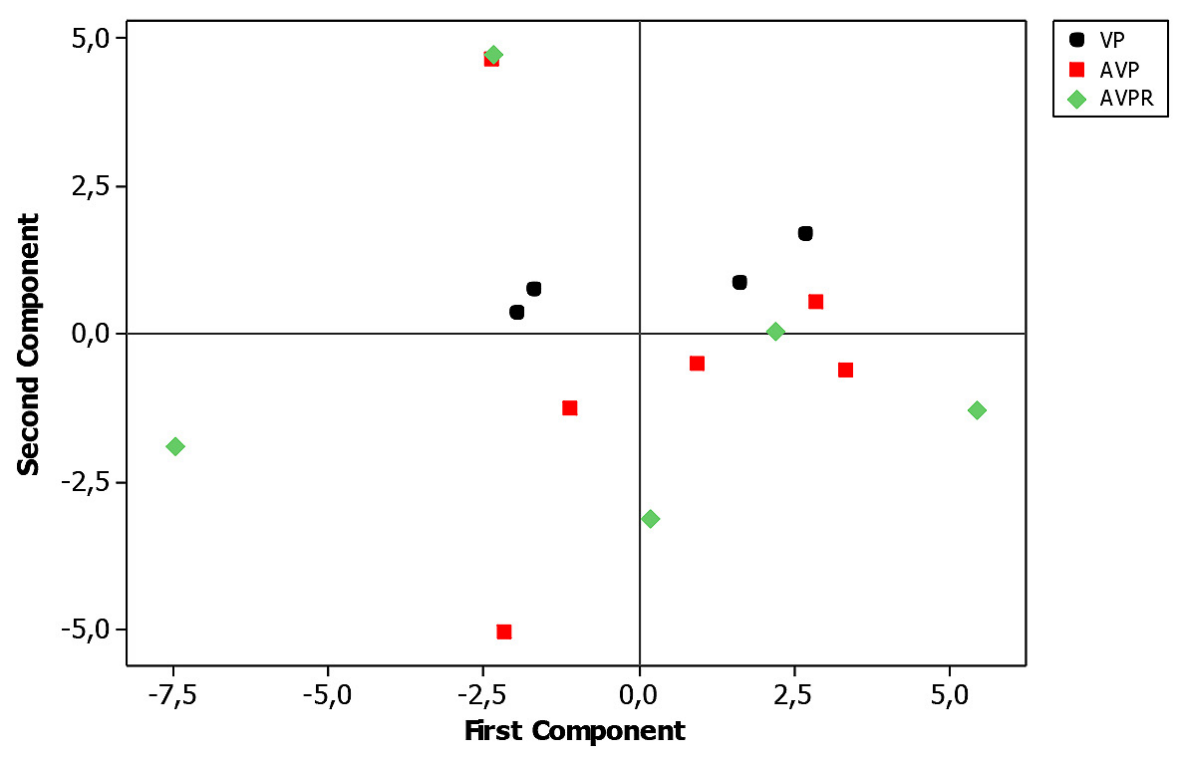
Principal component analysis of δ^13^C and δ^15^N values from experimental day zero to 46.

**Figure 4.**
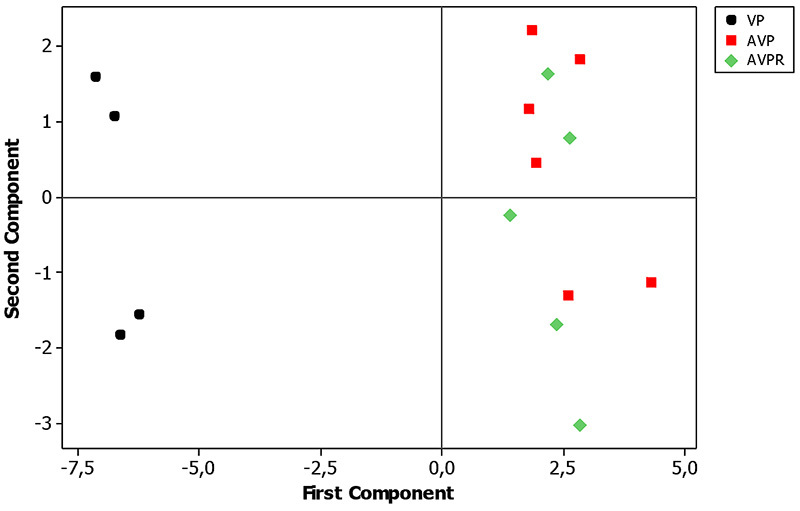
Principal component analysis of δ^13^C and δ^15^N values during experimental days 49 to 84.

**Figure 5.**
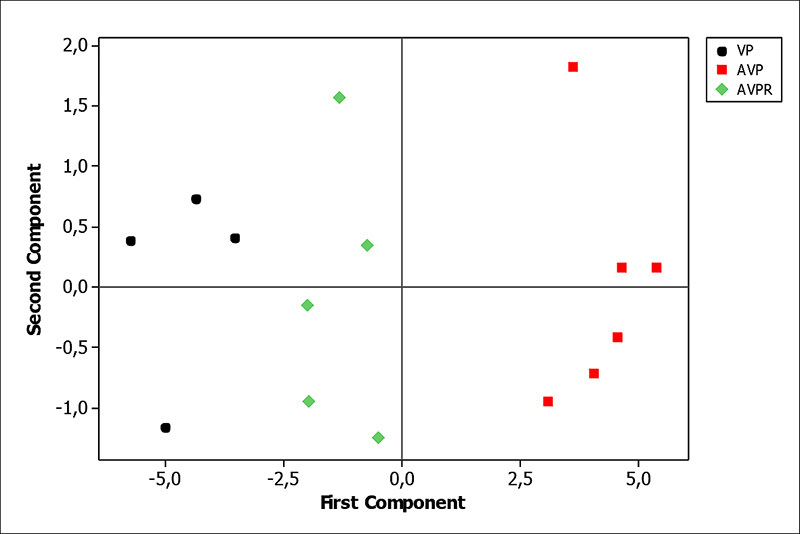
Principal component analysis of δ^13^C and δ^15^N values from experimental day 88 to 116.

The δ^13^C and δ^15^N variation among the experimental groups was detected in buffalo sera after 26 days of diet containing animal protein (p <0.01). It was observed for another 35 days in the AVPR group and maintained until the end of the experiment in the AVP group (p <0.01) (Figures 1 to 5).

AVPR did not differ from PV at the 88^th^ day but both groups differed from AVP, which demonstrates that when the strictly vegetable one replaces the animal protein diet, the isotopic signature of the animal protein diet is maintained until 41 days. Discrimination among groups had been most influenced by the ^13^C isotope (Figure 3, 4 and 5).

Total cell turnover or maximum incorporation of the diet, with MBM as a function of time in the AVPR group, for both ^13^C and ^15^N, occurred within approximately 60 days (Figures 1 and 2). AVPR and VP (control) groups presented similar isotopic values for the food profile at the 110^th^ day (Figure 1 and 2), showing that during this period the MBM diet was fully eliminated in the AVPR group.

PCA analysis revealed the stability of ^13^C and ^15^N isotopic values when compared with the behavior of diet incorporation as a function of time in the AVPR group, revealing stability of ^13^C and ^15^N isotopic values (Figures 1, 2 and 5).

The percentage of ^13^C atoms exchanged for incorporating the MBM diet in buffalo serum was 98.9% in AVP and 98.0% in AVPR, whereas that of ^15^N was 97.7% in AVP and 97.5% in AVPR. For diet elimination in the AVPR group, the atom percentage exchanged was 98.5% for ^13^C and 99.7% for ^15^N (Table 1). These results reveal reliability of the data because they present a small margin of error, which guarantees precision in the detection of animal protein in buffalo serum.

**Table 1.** Percentages of ^13^C and ^15^N atoms exchanged during incorporation and elimination of animal protein diet in the AVP and AVPR groups.

	^13^ **C atoms exchanged (%)**		^15^ **N atoms exchanged (%)**	
	**AVP**	**AVPR**	**AVP**	**AVPR**
Serum isotopic incorporation	98.9	98.0	97.7	97.5
Serum isotopic elimination	-	98.5	-	99.7

AVP: Animal and Vegetable Protein group; AVPR: Animal and Vegetable Protein Removal group.

An animal protein diet have already been successfully traced in other end products, where the MBM isotopic values found for laying egg yolk and egg albumen, was -17.40 ‰ and -17.24 ‰, respectively [27,28].

In the present study, the MBM diet, administered for 27 days, presented maximum incorporation at approximately 60 days, showing elimination at 63 days. Silva et al., determined the time periods for the incorporation and elimination of the diet by the detection of MBM using IRMS in sheep serum and plasma, followed by the evaluation of the turnover. It was observed that MBM took 54 days to be incorporated and 53 days to be eliminated from sheep serum, which demonstrates that traceability of biological samples by this technique, might reveal the animal protein diet [4].

The factors considered - namely age, growth rate, and body mass of the animals may influence the rates of incorporation and elimination of a diet [24,32], which could explain the longer time observed for buffalo serum samples. Future studies to evaluate turnover of ^13^C and ^15^N in different tissues of ruminants should be investigated.

## Conclusion

Zoonoses must be dealt with at the interface between human public health and veterinary public health. Prion diseases have caused not only great economic loss in the cattle industry of European countries but also provoked great public concern and put millions of people at risk and caused more than 160 human deaths [33].

Banning of supplemental feeding is generally considered a primary and necessary control strategy in an attempt to limit the transmission and spread of prion diseases [34]. Thus, the current study is particularly important because it shows, for the first time, that IRMS is sensitive and accurate for tracking the feeding profile of ruminants through blood analysis, thereby enabling investigation of the use of ABPs in ruminant feed worldwide.

### Abbreviations

Not applicable.
